# Casein Phosphopeptide-Amorphous Calcium Phosphate and Gallic Acid as a Complementary Approach for the Treatment of Early Enamel Lesions

**DOI:** 10.1155/ijbm/5536375

**Published:** 2025-11-25

**Authors:** Mahtab Mottaghi, Hossein Bagheri, Sara Majidinia, Abdolrasoul Rangrazi, Ali Faramarzi Garmroodi, Aliakbar Hodjatpanah Montazeri

**Affiliations:** ^1^School of Dentistry, Mashhad University of Medical Sciences, Mashhad, Iran; ^2^Dental Materials Research Center, Mashhad University of Medical Sciences, Mashhad, Iran; ^3^Department of Restorative and Cosmetic Dentistry, School of Dentistry, Mashhad University of Medical Sciences, Mashhad, Iran; ^4^Dental Research Center, Mashhad University of Medical Sciences, Mashhad, Iran; ^5^Independent Researcher, Mashhad, Iran

**Keywords:** CPP-ACP, gallic acid, microhardness, tooth remineralization

## Abstract

**Background:**

This study aimed to compare the effects of casein phosphopeptide-amorphous calcium phosphate (CPP-ACP) in combination with gallic acid (GA) compared to their individual effects on improving the microhardness of artificial enamel lesions at different depths from the surface.

**Materials and Methods:**

Forty enamel blocks were fabricated, with one-third of each block coated in varnish to establish a protected sound zone. The blocks were submerged in a demineralizing solution for 96 h to create artificial enamel lesions. Subsequently, half of the demineralized region was coated with varnish to create a reference demineralized area. The samples were allocated to four groups, with the remaining exposed portions treated with three experimental pastes (CPP-ACP paste, GA paste, and CPP-ACP + GA paste) and one control paste, representing the remineralized area. Vickers microhardness was assessed at depths of 20, 50, 100, 150, and 200 μm from the enamel surface in each region. Data were analyzed utilizing one-way ANOVA, followed by post hoc LSD tests. Cohen's d effect sizes and 95% confidence intervals (CIs) were also calculated to assess the strength of pairwise comparisons.

**Results:**

The CPP-ACP + GA paste exhibited the most significant enhancement in enamel microhardness at depths of 20, 50, and 100 μm with large effect sizes. While both CPP-ACP paste and GA paste significantly enhanced microhardness at 20 and 50 μm depths, they did not considerably impact microhardness at 100 μm depth.

**Conclusion:**

The combined use of CPP-ACP and GA showed a superior enhancement in the microhardness of artificial enamel lesions compared to the individual application of each agent.

## 1. Introduction

Dental caries, or tooth decay, is the world's most common oral health issue [1]. Its prevalence is rising in developing countries, mostly due to increased dietary intake of refined carbohydrates [2]. Dental caries is an ongoing process of tooth demineralization, ranging from asymptomatic subsurface lesions to clinically observable dentinal decay [3].

The demineralization-remineralization ratio describes the dynamic equilibrium between the loss and gain of minerals in tooth structure. Maintaining an optimal balance is crucial for sustaining tooth integrity and preventing dental caries [4]. When demineralization overcomes remineralization—frequently attributable to an improper diet, insufficient dental hygiene, and bacterial proliferation—enamel integrity is compromised, resulting in caries development. The acidic environment generated by cariogenic bacteria in the presence of fermentable carbohydrates contributes to demineralization [5].

In the past, tooth decay was known as an irreversible disorder resulting from acidogenic bacteria that caused the demineralization of dental structures. Dental caries is acknowledged as a reversible and dynamic phenomenon, where early enamel lesions, generally named white spot lesions, could be addressed with remineralizing agents [1, 6, 7]. The increasing focus on “minimally invasive dentistry” has increased interest in noninvasive treatments promoting enamel remineralization. The application of remineralizing agents on the tooth surface could restore mineral content and enhance enamel strength [5, 8].

Numerous chemicals have exhibited significant promise in facilitating the regeneration of hydroxyapatite (HAP) crystals, such as fluoride, amelogenin, casein phosphopeptide-amorphous calcium phosphate (CPP-ACP), resin infiltrants, tricalcium phosphate, and bioactive glass. These treatments provide a noninvasive method to address early enamel lesions, promoting the natural regeneration of HAP crystals without resorting to treatments like drilling or restorative fillings [1, 9].

CPP-ACP is a bioactive compound obtained from the milk protein casein and has been comprehensively evaluated for its ability to remineralize white spot lesions [6, 7]. CPP is a multi-phosphorylated peptide characterized by the sequence Ser (p)-Ser (p)-Ser (p)-Glu-Glu, where the phosphorylated serine residues facilitate the binding of calcium and phosphate ions, resulting in the formation of a stable ACP complex [5, 10]. Following application to the tooth surface, CPP-ACP nanocomplexes enhance the release of calcium and phosphate ions, establishing a supersaturated environment that aids in the reformation of HAP crystals crucial for enamel remineralization. This procedure strengthens the enamel and minimizes tooth sensitivity and erosion [10]. CPP-ACP demonstrates a major capacity for binding to diverse oral surfaces, such as the collected pellicle, dental plaque, soft tissues, and enamel HAP, hence increasing its application in dental care [5]. It is offered in many forms, including topical creams, toothpaste, and chewing gum products [11]. Moreover, several studies have integrated CPP-ACP into dental cement to augment their remineralization capabilities [12, 13]. Research has indicated that CPP-ACP–based tooth mousse can effectively reduce dentin hypersensitivity (DH) symptoms [14]. Nevertheless, its specific mechanism in facilitating intrafibrillar mineralization is yet to be fully elucidated [15].

*Galla chinensis*, a traditional Chinese medicine obtained from the Chinese sumac aphid (Baker) parasitizing *Rhus chinensis* Mill., has demonstrated the ability to improve enamel remineralization. Two distinct extracts from *Galla chinensis*, GCE-B1 and GCE-B2, have been characterized as gallic acid (GA) and methyl gallate [16].

GA (3,4,5-trihydroxybenzoic acid) is a natural polyphenol that demonstrates potential health advantages, including antioxidant and anticariogenic effects. GA affects the demineralization and remineralization of tooth hard tissues by binding to Ca^2+^ ions and stabilizing proteins. Further studies have shown that GA markedly enhances mineral deposition in simulated early enamel caries, predominantly in the surface layers [17].

Previous studies have shown the distinct remineralizing effects of CPP-ACP and GA on enamel caries [8, 16, 18, 19]. However, the combined impact of CPP-ACP and GA has not been comprehensively evaluated. Moreover, although several studies have assessed the remineralization efficacy of CPP-ACP and GA on WSLs, the results are inconclusive and contraindicatory [20–23]. Significantly, most of this research has concentrated exclusively on superficial alterations, neglecting the examination of remineralization at different lesion depths. Surface microhardness testing, which entails indentations on the outer enamel layer, is inadequate to detect underlying hardness changes or regional discrepancies within the lesion [24]. Depth-resolved microhardness testing has been utilized to evaluate subsurface alterations with greater precision [25]. To establish a thorough hardness profile, it is important to assess microhardness at specified intervals from the enamel surface [26].

This in vitro study aimed to assess the combined effect of CPP-ACP and GA, compared to their individual applications, on the remineralization of artificial enamel lesions at different depths from the surface.

## 2. Materials and Methods

**Figure 1:**
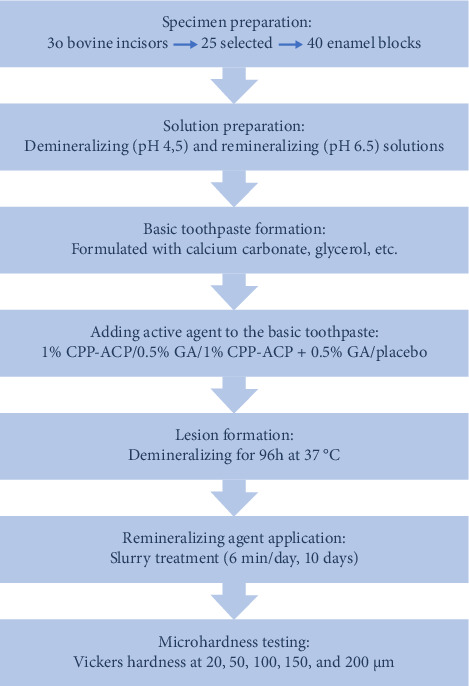
A workflow diagram summarizing the seven experimental steps of the in vitro research.

This in vitro experimental study was conducted in seven distinct phases: (I) specimen preparation, (II) solution preparation, (III) basic toothpaste formation, (IV) adding active agent to the basic toothpaste, (V) lesion formation, (VI) remineralizing agent application, and (VII) microhardness testing.  Figure 1 presents a schematic summary of the experimental steps.

The study protocol was approved by the Ethics Committee of Mashhad University of Medical Sciences (IR.MUMS.DENTISTRY.REC.1401.115). The remineralization efficacy of four synthetic toothpaste formulations on artificial enamel lesions was evaluated using Vickers microhardness testing at depths of 20, 50, 100, 150, and 200 μm.

The sample size was estimated based on a previous study [27]. Considering a statistical power of 80% and a significance level of 0.05—corresponding to a type I error (α) of 0.05 and a type II error (β) of 0.2—the study concluded that a minimum of 10 specimens per subgroup was required.

### 2.1. Specimen Preparation

Thirty freshly extracted bovine incisor teeth were obtained from a local slaughterhouse. Soft tissue remnants were removed utilizing a scaler (LM-Dental, Parainen, Finland). The teeth were subsequently preserved in a 0.1% thymol solution for 1 week, after which they were immersed in normal saline until initiating the experimental procedures.

**Figure 2:**
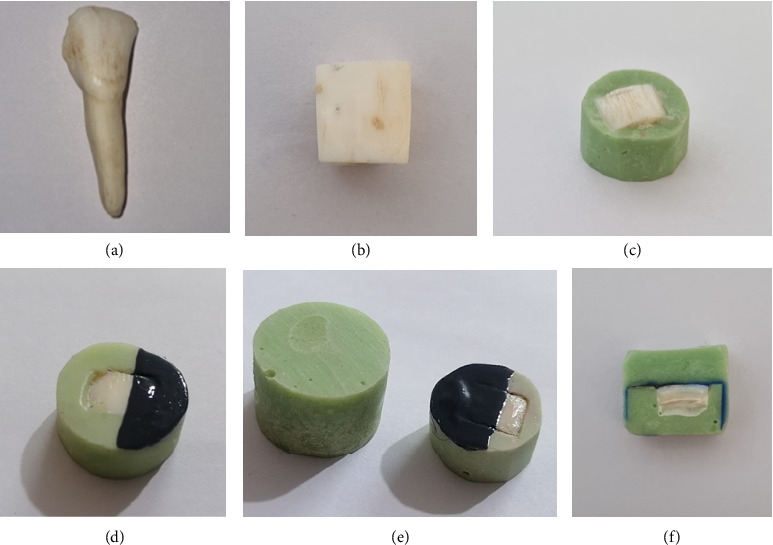
(a) Bovine incisor tooth; (b) the prepared enamel block; (c) a sample post-mounting in self-cure acrylic resin; (d) a sample after lesion formation; (e) a sample after applying the remineralizing toothpaste (right) and final mounting in self-cure acrylic resin (left); (f) samples prepared.

Before the experiment, the enamel surfaces were assessed using a stereomicroscope (Dino-Lite Pro, Anmo Electronics, New Taipei City, Taiwan) at × 10 magnification to eliminate any teeth showing cracks, fluorosis, hypoplasia, calcification, discoloration, deep grooves, or WSLs. Twenty-five sound teeth were selected (Figure 2(a)).

The crowns were separated from the roots using a water-cooled, low-speed, diamond-coated band saw (Isomet, Buehler, Lake Bluff, IL, USA). Enamel blocks were extracted from the flattest central region of the labial surface of each crown, with one or two blocks obtained per crown. Forty enamel blocks measuring 10 × 10 mm^2^ (width × length) were prepared (Figure 2(b)).

The labial surface of each block was coated with sticky wax and embedded in self-cure acrylic resin (Acropars, Kaveh, Tehran, Iran). Following the setting process, the specimens were removed from the molds, cleaned of any residual wax, and polished in succession with 800, 1200, 2500, 3000, and 5000 grit silicon carbide sandpapers (Starcke GmbH & Co. KG, Melle, Germany) while maintaining constant water irrigation to attain a mirror-like enamel surface (Figure 2(c)).

To establish a reference sound area for subsurface microhardness assessment, one-third of each enamel surface was coated with acid-resistant nail varnish (Figure 2(d)).

### 2.2. Solution Preparation

The demineralization and remineralization solutions were formulated with high-purity chemicals and distilled water. The demineralizing solution comprised 2.2 mM CaCl_2_, 2.2 mM Na_2_HPO_4_, and 0.05 M acetic acid, with the pH modified to 4.5 through the addition of NaOH [28].

The remineralizing solution, designed to mimic artificial saliva, consisted of 1 mM CaCl_2_, 3 mM Na_2_HPO_4_, and 100 mM NaCl, with a pH of 6.5 [29].

All chemical components were measured with a precision analytical balance, accurate to 0.0001 g (AND, Tokyo, Japan), and dissolved in distilled water using a magnetic stirrer to achieve homogeneity.

### 2.3. Basic Toothpaste Formation

A laboratory-formulated toothpaste was prepared at the Dental Materials Research Center, Mashhad University of Medical Sciences. The formulation consisted of 40% calcium carbonate (Merck, Darmstadt, Germany), 3% carboxymethyl cellulose (Sigma-Aldrich, St. Louis, USA), 30% glycerol (Merck, Darmstadt, Germany), 0.2% methylparaben (Merck, Darmstadt, Germany), 1.5% sodium lauryl sulfate (Merck, Darmstadt, Germany), and 25% distilled water [30].

CPP-ACP was synthesized at the Dental Materials Research Center, Dental School, Mashhad University of Medical Sciences [13].

Following the preparation of the basic toothpaste, three formulations of toothpaste were developed at the Dental Materials Research Center, Mashhad University of Medical Sciences, Mashhad, Iran. Each group was allocated 100 g of toothpaste. The four categories of toothpaste were as follows:

  Toothpaste containing 1% w/w CPP-ACP.
  Toothpaste containing 0.5% w/w GA (Exir, Tehran, Iran).
  Toothpaste containing 1% w/w CPP-ACP and 0.5% w/w GA.
  Placebo toothpaste without either CPP-ACP or GA (control group).

### 2.4. Lesion Formation

Artificial carious lesions were produced by immersing enamel blocks in a demineralizing solution at 37°C for a duration of 96 h. The solution was changed daily to ensure consistent demineralization conditions. After this period, chalky white regions resembling white spot lesions emerged on two-thirds of each block exposed to the solution. The enamel lesions generated by this protocol exhibit a depth ranging from 70 to 100 μm. The central one-third of each enamel surface was covered with acid-resistant nail varnish to function as a demineralized control area for the assessment of subsurface microhardness.

### 2.5. Remineralizing Agent Application

Following lesion formation, the specimens were coded and randomly allocated to four experimental groups according to the type of synthetic paste used. A toothpaste slurry was created by diluting each paste in distilled water at a 1:3 ratio and mixing with a magnetic stirrer for 5 min. The slurries were applied to the enamel surfaces for 6 min, after which they were rinsed with distilled water for 20 s. The treatment protocol was administered once daily for a duration of 10 days in a row. Specimens were stored in artificial saliva and incubated at 37°C between. Daily preparation of fresh slurry was conducted for each treatment session (Figure 2(e)).

### 2.6. Microhardness Testing

**Figure 3:**
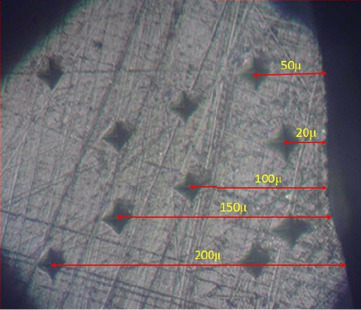
Vickers microhardness measurements at 20, 50, 100, 150, and 200 μm depths.

The microhardness test was conducted by remounting the samples in self-cure acrylic resin (Acropars, Kaveh, Tehran, Iran), ensuring full coverage of the enamel surfaces (Figure 2(e)). The specimens were subsequently sectioned to facilitate access to all three regions of interest (Figure 2(f)) and polished according to the previously outlined protocol. Microhardness was evaluated with a Vickers microhardness tester (KOOPA PAZHOOHESH, Iran; Model: MH3) at depths of 20, 50, 100, 150, and 200 μm (Figure 3). Depths were standardized utilizing a reference sample with calibrated indentations, and depth locations were recorded on the computer interface accordingly.

A load of 100 g was applied for 10 s at each measurement point. Two indentations were made per depth, and the average value was recorded as the microhardness for that layer.

To evaluate the remineralization potential among groups, the “Remin power” was computed at each depth utilizing the corresponding rREM and rDEM values according to the formulas mentioned below [29]:

  VHN: Vickers hardness number.
  DEM: Demineralized region.
  REM: Remineralized region.
  r_DEM = (VHN(DEM) − VHN(Sound))/(VHN(Sound)) × 100.
  r_REM = (VHN(REM) − VHN(Sound))/(VHN(Sound)) × 100.
  Remin power = r_REM-r_DEM.

### 2.7. Statistical Analysis

Data analysis was conducted using SPSS software, Version 22 (SPSS Inc., IBM Corp., Armonk, NY, USA), with a significance level set at *p* < 0.05. Descriptive data were presented as means and standard deviations for the determined Remin power (%) at each depth across all groups. To examine the baseline consistency of intact enamel microhardness, a one-way ANOVA was done at a depth of 20 μm. The primary analysis aimed to determine the variations in remineralization potential across the four experimental groups. For this purpose, separate one-way ANOVAs were performed at each enamel depth (20, 50, 100, 150, and 200 μm), followed by post hoc pairwise comparisons using the least significant difference (LSD) test to discover statistically significant differences between groups.

The sample size was estimated based on a previous study. Considering a statistical power of 80% and a significance level of 0.05—corresponding to a type I error (α) of 0.05 and a type II error (β) of 0.2—the study concluded that a minimum of 10 specimens per subgroup was required.

## 3. Results

**Figure 4:**
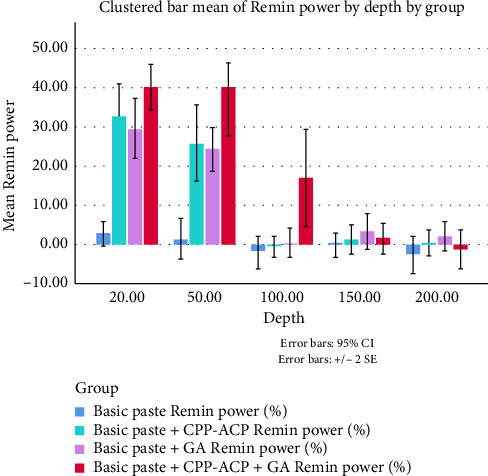
Remin power (%) of the four groups at depths of 20, 50, 100, 150, and 200 μm.

A depth of 20 μm was used to evaluate the uniformity of the sound enamel among the groups, as shown in Table 1. One-way ANOVA analysis indicated no statistically significant variations in the microhardness values of the enamel blocks (*p* = 0.94). The microhardness values of the groups at different depths are shown in Table 2. The findings of the Remin power study are presented in Table 3 and Figure 4. Table 3 demonstrates that the control group consistently displayed the lowest mean Remin power at all assessed depths. The group treated with the CPP-ACP + GA paste had the greatest mean Remin power values at 20, 50, and 100 μm depths.

The results of the post hoc LSD test, which compares the mean Remin power values across the groups at depths of 20, 50, and 100 μm, are detailed in Tables 4, 5, and 6. Statistically significant differences were noted between the control and treatment groups at depths of 20 and 50 μm. At a depth of 100 μm, no notable differences were observed between the CPP-ACP, GA, and control groups. The CPP-ACP + GA group demonstrated a markedly enhanced remineralization capability at this depth compared to the other groups.

The data in Tables 4, 5, and 6 demonstrate that the control group consistently showed the lowest mean Remin power at 20 and 50 μm, with statistically significant differences observed compared to all treatment groups (*p* < 0.001).

At 20 μm (Table 4), the CPP-ACP + GA paste demonstrated the greatest remineralization potential, as indicated by microhardness measurements. A notable difference was observed compared to the GA group (*p*=0.0255), while the difference with the CPP-ACP group was not statistically significant (*p*=0.117).

At 50 μm (Table 5), the CPP-ACP + GA group exhibited the highest remineralization power values, significantly above the samples in the CPP-ACP (*p*=0.046) and GA (*p*=0.024) groups.

Table 6 indicates no statistically significant differences at 100 μm among the control, CPP-ACP, and GA groups. In contrast, the CPP-ACP + GA paste exhibited significantly higher values compared to the control (*p* < 0.001), CPP-ACP (*p*=0.001), and GA (*p*=0.002) groups.

The identified remineralization patterns are further supported by the effect size (Cohen's d) analysis presented in Table 7. At a depth of 20 μm, large effects were observed when comparing treatment groups with the base group: CPP-ACP + GA (*d* = 5.143, 95% confidence interval [CI] [3.324–6.962]), CPP-ACP (*d* = 3.186, 95% CI [1.865–4.506]), and GA (*d* = 2.942, 95% CI [1.677–4.206]). The difference between GA and CPP-ACP was minimal (*d* = 0.266, 95% CI [−0.615–1.146]).

At 50 μm, GA and CPP-ACP + GA showed a moderate difference (*d* = 1.054, 95% CI [0.118–1.989]), while CPP-ACP + GA vs. base (*d* = 3.022, 95% CI [1.739–4.304]) and GA vs. base (*d* = 2.736, 95% CI [1.516–3.955]) indicated large effects.

At 100 μm, CPP-ACP + GA maintained its superiority, showing medium-to-large effect sizes compared to base (*d* = 1.395, 95% CI [0.417–2.372]), CPP-ACP (*d* = 1.238, 95% CI [0.281–2.194]), and GA (*d* = 1.163, 95% CI [0.215–2.111]).

In deeper sections (150–200 μm), effect sizes were generally small and statistically nonsignificant, with most 95% CIs crossing zero.

These results highlight the consistent advantage of CPP-ACP + GA in promoting enamel remineralization across different depths.

## 4. Discussion

The main goal of modern dentistry is the noninvasive treatment of incipient caries, which is achievable through the enhancement of enamel remineralization. Natural substances are frequently preferred over other existing remineralizing agents because of their affordability, efficacy, and safety [31].

Various methodologies have been used to evaluate enamel remineralization, including visual examination, quantitative light-induced fluorescence, polarizing microscopy, microradiography, and microhardness assessments. Since enamel hardness is associated with mineral content and remineralization is indicated by mineral gain, the microhardness test is commonly used to measure enamel remineralization [32]. This study employed Vickers microhardness tests to evaluate the remineralization capability of several agents at different lesion depths. By typical practices for depth-specific enamel microhardness research, the sectioned cross-sectional surfaces were polished to achieve flat, homogeneous surfaces that facilitate precise and reproducible indentations. This method aligns with other research that highlights the importance of preparation in preventing artifacts and variability [33–35]. All groups underwent identical procedures for sectioning and polishing to maintain experimental consistency and comparability.

We evaluated the effects of CPP-ACP and GA, which are naturally derived compounds. CPP-ACP, extracted from milk proteins, promotes remineralization by creating a supersaturated condition of calcium and phosphate ions on the tooth surface [36]. CPP-ACP nanocomplexes diminish surface roughness and enhance the micromorphological characteristics of demineralized enamel [37]. Previous studies have established that CPP-ACP enhances surface microhardness [1, 4, 8, 38], and some investigations, including Reynolds' investigation, indicate its potential to infiltrate underlying enamel lesions [39].

In the current study, CPP-ACP markedly enhanced enamel microhardness at depths of 20 and 50 μm but not at 100 μm. This aligns with the results from Miyahira et al. [19] and Oliveira et al. [40], which showed the efficacy of CPP-ACP up to 75 μm. Larson et al. [41] proposed that the initial phase of surface remineralization decreases ion permeability, consequently preventing the diffusion of ions into the deeper layers of enamel.

GA, a natural polyphenol produced from *Galla chinensis*, has been demonstrated to suppress enamel demineralization and modify HAP production via calcium ion interactions [16, 18, 42]. Its antimicrobial activities specifically target cariogenic bacteria, including *Streptococcus mutans* and *Lactobacillus*. Our data indicate that GA enhanced enamel microhardness at 20 and 50 μm, supporting previous research documenting its remineralizing capabilities [2, 17].

Due to the complexity of *Galla chinensis*'s composition, we assessed GA separately. Interactions between enamel matrix proteins and GA across varying pH levels may further reduce demineralization [43]. Furthermore, GA's capacity to interact with milk proteins and calcium ions could result in the precipitation and stability of active drugs [44].

Our findings also indicated that the remineralization efficacy of CPP-ACP paste surpassed that of GA paste at the 20 and 50 μm depths; however, this discrepancy did not attain statistical significance. The comparable remineralization effects noted in the GA groups may be attributed to the multifactorial mechanism of action of GA. GA interacts with calcium ions to facilitate HAP production and prevent demineralization, as seen by the recovery of surface microhardness in enamel lesions treated with GA [45, 46]. It also interacts with enamel matrix proteins, increasing mineral affinity at the lesion interface [46]. Several earlier research studies demonstrated the effectiveness of CPP-ACP in promoting enamel remineralization [21, 22, 47]. GA is an active constituent of grape seed extract. Some research indicates that grape seed extract demonstrates superior remineralization capacity compared with CPP-ACP [20, 23]. Discrepancies in results could be related to changes in research design, encompassing differences in methodology, treatment durations, and the stage or severity of WSLs [8]. Rafiei et al. [48] indicated that CPP-ACP might function as a substitute for fluoride in daily-use toothpaste to enhance enamel remineralization.

Enamelin, tuftelin, and sheathlin are essential proteins in mature enamel structures. The interactions of these proteins with GA across different pH levels may lead to decreased demineralization. Furthermore, polyphenols like GA can modify milk proteins' structural and functional characteristics, with casein exhibiting a notably high binding affinity [44]. GA has the ability to interact with milk proteins [47] and can also cross-link with calcium ions, leading to the formation of precipitates [48]. The interactions indicate that GA may stabilize the CPP-ACP complex through cross-linked network formation, which enhances structural integrity and extends the release of calcium and phosphate ions at the enamel surface. This stabilization likely preserves a supersaturated environment, facilitating continuing remineralization, even within deeper enamel layers. Mao et al. demonstrated that polyphenol–protein binding enhances the stability of CPP-ACP nanocomplexes [49], which extends ion availability at the lesion interface and improves remineralization [44, 50]. The observed improvement in microhardness at a depth of 100 μm when CPP-ACP and GA are used in combination may be attributed to this mechanism, in contrast to the limited effects noted with each agent alone. The findings indicate that the combined use of CPP-ACP and GA produced the highest microhardness values at depths of 20 and 50 μm, with a significant increase observed at 100 μm, a depth minimally affected by the individual agents. Our findings indicate that GA enhances the structural performance of CPP-ACP while also improving its bioavailability and penetration into subsurface enamel lesions.

Some variations in microhardness data were observed, especially at greater enamel depths, aligning with results from prior enamel remineralization investigations utilizing bovine teeth [51, 52]. The biological variability of bovine enamel, differences in lesion depth despite consistent methods, and variations in agent penetration between layers may all contribute to the reported standard deviations. The statistically significant results at 20, 50, and 100 μm demonstrate consistent patterns across samples and validate the efficacy of the investigated formulations.

The statistically substantial enhancement in enamel microhardness found in the combined CPP-ACP and GA formulation across various lesion depths holds major therapeutic implications. This combination may function as an efficient noninvasive treatment for early enamel carious lesions, facilitating deeper remineralization that exceeds the efficacy of individual treatments. Clinically, this formulation may mitigate lesion progression, prevent or minimize the necessity for restorative treatment, and facilitate minimally invasive dentistry strategies focused on conserving natural tooth structure. These findings establish a promising basis for future clinical trials and the advancement of improved remineralizing dental approaches.

The principal limitations of this in vitro study are the use of artificial saliva, bovine teeth, artificially created enamel lesions, and the short-term assessment of the investigation protocol. Although this in vitro study did not comprehensively represent the oral environment, from another perspective, it provided an advantage in controlling potential confounding variables. However, it is essential to recognize the physicochemical disparities between bovine and human teeth, especially regarding enamel structure and porosity. Bovine enamel exhibits a reduced fluoride concentration and increased porosity [53]. Human enamel exhibited larger crystallite sizes and reduced microstrain relative to bovine enamel. An elevated microstrain value indicates reduced crystallinity and an increased specific surface area of the sample [54]. Ankily et al. [55] demonstrated insignificant differences in structure, chemical composition, and radiographic density between human and bovine hard tissues, thereby justifying using bovine teeth as an alternative to human teeth in dental research. Schmidlin et al. [52] recommended using bovine teeth instead of human teeth, highlighting advantages such as enhanced accessibility, more consistent composition, and increased size. These characteristics facilitate the production of many enamel blocks from one tooth, thus reducing intersample variability. Thereby, the advantages of utilizing bovine teeth overcome the drawbacks linked to the lack of human specimens. Another limitation of this study is the lack of microstructural analysis. Our findings indicate an improved remineralization effect from the combined therapy; however, the conclusions are exclusively derived from Vickers microhardness measurements. Microstructural analyses, including scanning electron microscopy (SEM) and transmission electron microscopy (TEM), were excluded due to limitations in study scope, time constraints, and a focus on nondestructive testing methods. Consequently, the identified trends must be analyzed in relation to these methodological constraints. This work included graphical representations of microhardness measures at different lesion depths to enhance data visualization and transparency. A methodological limitation is the mechanical polishing of enamel specimens, which eliminates the superficial aprismatic enamel layer. This process enhances surface uniformity and reproducibility for microhardness testing [56, 57]; however, it may not fully reflect the natural oral surface conditions present during in vivo remineralization.

The CPP-ACP and GA combination has been shown to increase microhardness values; therefore, this formulation may be recommended for clinical use as a toothpaste for daily use or as a professional topical therapy for the early management of enamel lesions. It may be especially beneficial for high-risk patients, such as those undergoing orthodontic treatment or receiving bleaching treatments. However, more preclinical and clinical studies with the long-term reliability of the suggested treatment strategy are essential to assess the effectiveness of CPP-ACP and GA as remineralizing agents.

## 5. Conclusion

The findings from Vickers microhardness measurements lead to the following conclusions:

• The combined application of CPP-ACP and GA improved microhardness values at all evaluated lesion depths. This combination exhibited the greatest enamel microhardness enhancement, with a notable improvement at a depth of 100 μm, which was not found with the individual agents.
• When applied independently, CPP-ACP and GA significantly enhanced enamel microhardness to a depth of 50 μm. No statistically significant difference was found between their effects at these depths.

The findings indicate that the combined application of CPP-ACP and GA may improve remineralization potential compared to their individual use, as evidenced by microhardness results. Additional microstructural studies are necessary to validate these effects and clarify the underlying mechanisms.

## Figures and Tables

**Table 1:** The microhardness values at the depth of 20 μm.

Depth (μ)	Groups	*N*	*p* value
20	Base	10	0.94
	CPP-ACP	10	
	GA	10	
	CPP-ACP + GA	10	

**Table 2:** The microhardness values of the groups at different depths.

Groups	*N*	Enamel depth (μm)	Sound enamel	Remin power (%)	r_DEM_	r_REM_
Group Ι	10	20	305.09 ± 35.34	2.49	−53.84 ± 9.70	−51.34 ± 8.82
		50	328.06 ± 17.71	1.26	−28.17 ± 17.07	−26.91 ± 13.90
		100	350.64 ± 47.44	−2.01	−3.32 ± 12.34	−5.33 ± 12.00
		150	372.46 ± 24.41	−0.21	−1.28 ± 6.64	−1.50 ± 8.43
		200	352.32 ± 30.68	−2.75	2.46 ± 8.12	−0.29 ± 5.81

Group ΙΙ	10	20	290.35 ± 32.38	32.73	−54.42 ± 8.39	−21.68 ± 14.57
		50	323.42 ± 33.26	25.79	−37.84 ± 12.33	−12.05 ± 13.77
		100	347.01 ± 39.18	−0.63	−10.33 ± 14.29	−10.97 ± 13.06
		150	342.73 ± 36.67	1.11	0.04 ± 6.46	1.16 ± 5.13
		200	367.92 ± 26.54	0.28	−2.81 ± 7.61	−2.52 ± 7.39

Group ΙΙΙ	10	20	300.03 ± 19.09	29.48	−56.89 ± 10.30	−27.41 ± 11.90
		50	342.42 ± 35.19	24.23	−33.42 ± 13.08	−9.18 ± 11.48
		100	337.97 ± 34.11	0.14	−2.45 ± 13.80	−2.31 ± 12.72
		150	354.98 ± 28.11	3.33	−3.80 ± 8.63	−0.47 ± 8.73
		200	366.9 ± 34.92	2.09	−3.95 ± 9.44	−1.86 ± 7.74

Group ΙV	10	20	289.53 ± 27.26	39.98	−55.50 ± 11.50	−15.52 ± 12.27
		50	327.88 ± 44.46	37.03	−44.21 ± 11.64	−7.18 ± 13.16
		100	339.49 ± 36.74	16.87	−20.62 ± 17.51	−3.75 ± 8.29
		150	369.08 ± 30.13	1.39	−7.10 ± 9.49	−5.71 ± 9.41
		200	371.15 ± 30.28	−1.52	−2.10 ± 8.51	−3.63 ± 6.33

**Table 3:** Remin power values based on different depths.

Depth (μ)	Groups	*N*	Mean	Std. deviation	Std. error	*p* value
20	Base	10	2.496	4.923	1.556	< 0.001
	CPP-ACP	10	32.738	12.49	3.949	
	GA	10	29.482	12.003	3.795	
	CPP-ACP + GA	10	39.98	9.056	2.863	

50	Base	10	1.26	7.946	2.513	< 0.001
	CPP-ACP	10	25.795	15.306	4.84	
	GA	10	24.236	8.827	2.791	
	CPP-ACP + GA	10	37.036	14.739	4.66	

100	Base	10	−4.166	8.667	2.74	0.001
	CPP-ACP	10	−0.634	4.488	1.419	
	GA	10	0.141	5.815	1.838	
	CPP-ACP + GA	10	16.87	19.493	6.164	

150	Base	10	−0.215	5.185	1.639	0.649
	CPP-ACP	10	1.115	6.042	1.91	
	GA	10	3.336	7.234	2.287	
	CPP-ACP + GA	10	1.393	6.277	1.985	

200	Base	10	−2.045	6.483	2.05	0.471
	CPP-ACP	10	0.287	5.295	1.674	
	GA	10	2.09	5.819	1.84	
	CPP-ACP + GA	10	−1.527	7.782	2.461	

*Note:* The mean difference is significant at the 0.05 level.

**Table 4:** The LSD post hoc test analysis at a depth of 20 μm.

Depth (μ)	(I) paste	(J) paste	Mean difference (I-J)	*p* value
20	Base	CPP-ACP	−30.242^∗^	< 0.001
	Base	GA	−26.986^∗^	< 0.001
	Base	CPP-ACP + GA	−37.484^∗^	< 0.001
	CPP-ACP	GA	3.255	0.475
	CPP-ACP	CPP-ACP + GA	−7.242	0.117
	GA	CPP-ACP + GA	−10.498^∗^	0.0255

^∗^The mean difference is significant at the 0.05 level.

**Table 5:** The LSD post hoc test analysis at a depth of 50 μm.

Depth (μ)	(I) paste	(J) paste	Mean difference (I-J)	*p* value
50	Base	CPP-ACP	−24.534^∗^	< 0.001
	Base	GA	−22.975^∗^	< 0.001
	Base	CPP-ACP + GA	−35.775^∗^	< 0.001
	CPP-ACP	GA	1.558	0.776
	CPP-ACP	CPP-ACP + GA	−11.240^∗^	0.046
	GA	CPP-ACP + GA	−12.799^∗^	0.024

^∗^The mean difference is significant at the 0.05 level.

**Table 6:** The LSD post hoc test analysis at a depth of 100 μm.

Depth (μ)	(I) paste	(J) paste	Mean difference (I-J)	*p* value
100	Base	CPP-ACP	−3.531	0.488
	Base	GA	−4.308	0.399
	Base	CPP-ACP + GA	−21.036^∗^	< 0.001
	CPP-ACP	GA	−0.776	0.879
	CPP-ACP	CPP-ACP + GA	−17.504^∗^	0.001
	GA	CPP-ACP + GA	−16.728^∗^	0.002

^∗^The mean difference is significant at the 0.05 level.

**Table 7:** Cohen's d effect sizes and 95% CIs to compare enamel microhardness at different depths.

Depth (μm)	(I) paste	(J) paste	Cohen's d	95% CI
20	Base	CPP-ACP	3.186	1.865–4.506
	Base	GA	2.942	1.677–4.206
	Base	CPP-ACP + GA	5.143	3.324–6.962
	CPP-ACP	GA	0.266	−0.615–1.146
	CPP-ACP	CPP-ACP + GA	0.664	−0.237–1.564
	GA	CPP-ACP + GA	0.987	0.059–1.916

50	Base	CPP-ACP	2.012	0.936–3.088
	Base	GA	2.736	1.516–3.955
	Base	CPP-ACP + GA	3.022	1.739–4.304
	CPP-ACP	GA	0.125	−0.753–1.002
	CPP-ACP	CPP-ACP + GA	0.748	−0.159–1.655
	GA	CPP-ACP + GA	1.054	0.118–1.989

100	Base	CPP-ACP	0.512	−0.379–1.403
	Base	GA	0.584	−0.311–1.479
	Base	CPP-ACP + GA	1.395	0.417–2.372
	CPP-ACP	GA	0.149	−0.729–1.027
	CPP-ACP	CPP-ACP + GA	1.238	0.281–2.194
	GA	CPP-ACP + GA	1.163	0.215–2.111

150	Base	CPP-ACP	0.236	−0.643–1.116
	Base	GA	0.564	−0.33–1.458
	Base	CPP-ACP + GA	0.279	−0.601–1.16
	CPP-ACP	GA	0.333	−0.549–1.216
	CPP-ACP	CPP-ACP + GA	0.045	−0.832–0.922
	GA	CPP-ACP + GA	0.287	−0.594–1.168

200	Base	CPP-ACP	0.394	−0.491–1.279
	Base	GA	0.671	−0.23–1.572
	Base	CPP-ACP + GA	0.072	−0.804–0.949
	CPP-ACP	GA	0.324	−0.558–1.206
	CPP-ACP	CPP-ACP + GA	0.273	−0.608–1.153
	GA	CPP-ACP + GA	0.526	−0.365–1.418

## Data Availability

Data are contained within the article.
